# Prevalence and Predictors of Home Use of Glucometers in Diabetic Patients

**DOI:** 10.7759/cureus.1330

**Published:** 2017-06-10

**Authors:** Syed A Farhan, Ali T Shaikh, Maria Zia, Bilal R Kahara, Ramsha Muneer, Muzna Rehman, Ayesha Mubashir, Hassaan Sadiq, Durr-e-Amna Siddiqui, Syed M Haseeb, Hafsa Tanveer, Khadijah Siddiq, Saib B Mujtaba, Samir A Mirza, Hira Feroz, Kaneez Fatima

**Affiliations:** 1 Civil Hospital, Dow University of Health Sciences (DUHS), Karachi, Pakistan; 2 Department of Internal Medicine, Dow University of Health Sciences (DUHS), Karachi, Pakistan; 3 Dow Medical College, Dow University of Health Sciences (DUHS), Karachi, Pakistan

**Keywords:** self monitoring, predictors, diabetes mellitus, home glucometer, smbg

## Abstract

**Background:**

Self-monitoring of blood glucose (SMBG) is a critical component of diabetes care. However, it has been shown that use of glucometers in developing countries such as Pakistan is limited. The aim of this study was to determine the frequency of glucometer usage in the urban diabetic population of Karachi and to identify variables that influenced the likelihood of practice of SMBG.

**Methods:**

A cross-sectional study was conducted among 567 adult diabetic patients selected at random from the out-patient departments of multiple healthcare institutions in Karachi categorized into two settings; Government and Private. Non-diabetics, patients having gestational diabetes, diabetes insipidus and Cushing’s syndrome and terminally ill patients were excluded. Pearson Chi-square and Mann-Whitney U test were applied as the primary statistical method.

**Results:**

Prevalence of home glucometer usage was 59% (n= 331). High socioeconomic status (*p* < 0.001), receiving care from private institutions (*p* < 0.001), higher education (*p* < 0.001), a family history of diabetes (*p *=0.001), awareness regarding diabetes (*p* < 0.001), having diabetes for > five years (*p *<0.001), and managing diabetes via pharmacological interventions (*p *<0.001) (versus diet and exercise) were significant positive predictors of glucometer usage.

**Conclusions:**

Our study demonstrates the increasing trend in use of SMBG. Lack of awareness and cost of glucometers were reported to be the main reasons for not practicing SMBG. Given these factors are easily modifiable, government subsidized initiatives and awareness programs can result in a successful public health strategy to promote SMBG.

## Introduction

According to a survey conducted by the International Diabetes Federation (IDF), 415 million people globally were diagnosed with diabetes mellitus (DM) in the year 2015 [1]. In 2016, the World Health Organization (WHO) estimated the prevalence of DM in Pakistan to be 9.8% which is expected to increase in the near future [2]. Diabetes can cause multi-system complications including retinopathies, nephropathies, neuropathies, foot ulcers, ischemic heart disease and stroke [3-4]. Self-monitoring of blood glucose (SMBG) via the aid of portable glucometers used by patients at home comprises one of the major components of diabetes management. The aim of these techniques is to allow diabetic patients to establish greater glycemic control which has been shown to decrease long-term macro- and micro-vascular complications of diabetes [5].

Testing of glycated hemoglobin (HbA1c) levels remains the gold standard for monitoring glycemic control, however, it has some limitations. Because it is performed once every three months, it fails to provide information regarding day to day and intra-day fluctuations. In contrast, SMBG techniques allow for greater scrutinizing of daily glucose fluctuations in the affected patient including fasting, pre- and post-prandial glucose levels. This provides patients with immediate feedback regarding the effects of their lifestyle, physical activities or food choices on their glucose levels and providing adequate information to the health-care practitioners for adjustment of therapy [6]. For these reasons, SMBG is becoming an ever more popular adjunct to traditional HbA1c testing. Currently, SMBG techniques are more widely utilized in more developed economies, for example, Germany or the United States of America (USA), in comparison to developing countries such as Malaysia, India or Pakistan [7]. Studies show that SMBG is efficacious in view of helping patients regulate HbA1C levels [8-11], though the evidence is conflicting [12-13]. Nevertheless, SMBG has been deemed as a critical component of diabetes care by the American Diabetes Association [14].

Urbanization, with its associated changes in diet and lifestyle, has been found to significantly contribute to the development of diabetes mellitus in the population [15]. As Pakistan’s largest urban agglomeration, Karachi provides a window into the ever enlarging prevalence of this disease in Pakistani cities. In light of sharply climbing statistics, it is essential to find the prevalence of glucometer usage in the population, especially with the advent of research that shows that people of South Asian ancestry such as Pakistanis are relatively more prone to diabetes than people of other races [16]. This information can be utilized to help patients make better lifestyle and pharmacological choices thereby aiding them in better management of their disease. Our study aims to determine the proportion of the urban diabetic population in Karachi that practices home-based SMBG and to determine the factors that influence the likelihood of glucometer utilization.

## Materials and methods

This cross-sectional study was carried out amongst the diabetic population of Karachi. Diabetic patients were selected at random from the out-patient departments of two different hospital settings, Government and Private. Data were collected from multiple hospitals to ensure equal representation of all socioeconomic statuses within an urban and semi-urban population. The study was conducted over a time period of three months from January to March 2017 after approval from the Institutional Review Board of Dow University of Health Sciences. Individuals considered eligible were both established Type-2 diabetics and residents of Karachi having had the disease for at least six months. Non-diabetics, patients having gestational diabetes, diabetes insipidus or Cushing’s syndrome were excluded. Terminally ill patients were also excluded. A structured questionnaire was compiled and was thoroughly reviewed by two proficient doctors for relevance, coherence, and clarity. The questionnaire adequately addressed our research objective and the questions focused on the extraction of essential information from recent events like frequency of usage of self-monitoring of blood glucose (SMBG) per week and timing of self-monitoring of blood glucose. This was further verified by the help of proxy sources such as log books and close attendants, which helped in reducing recall bias. A standard protocol was used for interviewing to eliminate interviewer bias. The questionnaire was then pilot tested to reduce obscurity. A consent form was signed by every participant and the questionnaire was translated into a local language to reduce linguistic barriers. Out of the 600 patients approached, 567 completed the questionnaire fully. Thus, the co-operation rate of the tested sample was 94.5%. The remaining 33 questionnaires were discarded due to inadequate and incomplete data. No imputation method was utilized.

The questionnaire was divided into three sections, the first section comprised of demographic variables such as age, gender, ethnicity, marital and socioeconomic status. Participants with a monthly income of ≥ Pakistani rupee 60,000 were categorized as high socioeconomic status and < Pakistani Rupee 60,000 as low. The level of education was classified into two groups; uneducated/primary education was defined as having attended grades one-five, while secondary education was defined as having attended grades six-12 or higher studies comprised of tertiary and quaternary education (university, graduates or masters). The second section was regarding characteristics of the disease. This included the duration of diabetes, which was recorded as; ≤ 5 years and > 5 years. Patient’s last visit to the doctor was recorded as; ≤ 3 months and > 3 months. Both lifestyle modification and type of pharmacological treatment used was taken into consideration. The participants’ last HBA1c levels were also noted by checking their blood test reports. Uncontrolled diabetes was defined as having an HbA1c value of > 6.5%. Patients were also queried regarding comorbidities and diabetic complications. The third section focused primarily on the use of SMBG, its duration of use, the frequency of use per week and if its use caused any impact on the patient’s life. Patients who did not use SMBG were asked reasons for not doing so.

Categorical data was presented as frequency and percentages while continuous data was presented in terms of mean and standard deviations. Pearson Chi-square was applied to test for differences between categorical variables. Mann-Whitney U was applied to test for difference between non-parametric data for continuous and categorical variables. *The P* value < 0.05 was considered significant in all cases. Data were entered and analyzed descriptively using IBM Statistical Package for the Social Sciences (IBM SPSS Statistics for Windows, Version 20.0. Armonk, New York). Tables were constructed using Microsoft Excel 2016.

## Results

### Characteristics of patients

A total of 567 patients with diabetes were enrolled in the study. Of 48.7% (n=276) of the study population was taken from government hospitals. Mean age of the participants was 51.8 years (SD=12.1). Of the population surveyed, females constituted a majority of 59.6% (n=338) and 94.2% (n=534) of the participants were married and 86.9% (n=493) were reportedly non-smokers. Nearly 12.7% (n=72) of the population belonged to high socioeconomic status and 57.3% (n=325) of the participants had received secondary education or above. In which, 55% (n=312) of the population reported having diabetes for > 5 years. Table 1 shows the demographic and clinical characteristics of the study population.

**Table 1:** Demographic and clinical characteristics of the study population

CHARACTERISTICS	N (%)
Age	
10-50 Years	263 (46.4%)
51-95 Years	304 (53.6%)
Gender	
Male	229 (40.4)
Female	338 (59.6)
Marital Status	
Married	534 (94.2)
Others	33 (5.8)
Education level	
Uneducated/ Primary education (grade 1-5)	242 (42.7)
Secondary/Higher Education (grade 6-12/ graduate)	325 (57.3)
Socio-economic status	
High (> 60,000 PKR)	72 (12.7)
Low (< 60,000 PKR)	495 (87.3)
Duration of Diabetes	
< 5 years	225 (44.9)
> 5 years	324 (60.3)
Hyperlipidemia	140 (24.7)
Hypertension	308 (54.3)
Family history of diabetes	357 (62.9)
Complications of diabetes	331 (58.4)
Medications	
Hypoglycemic tablets	362 (63.8)
Insulin injections	65 (11.5)
Both tablets and insulin injections	115 (20.3)

The study covered patients undergoing all treatment regimens; diet and exercise (n=24, 4.2%), oral hypoglycemics (n=362, 63.8%), insulin (n=65, 11.5%) and both oral hypoglycemics and insulin (n=115, 20.3%). The HbA1c results for the last three months were available for 122 patients and only 19.7% had controlled diabetes (HbA1c ≤ 6.5%). Mean HbA1c was 8.33% (standard deviation SD=2.52).

### Self-monitoring of blood glucose usage

Of all the participants, 59% (n=331) used home glucometers. Mean frequency of glucometer use per week was 3.0 times (SD=3.92). Participants who performed SMBG (n=332, 59%) received awareness regarding glucometers mainly through hospitals/awareness programs (n=209, 63%), friends and relatives (n=110, 33%). Those who did not perform SMBG majorly gave high expense of glucometers (n=108, 46%), no awareness regarding them (n=55, 24%) and poor education about its usage (n=50, 21%) as reasoning. The discomfort of use only affected a small minority (n=20, 9%).

As shown in Table 2, results of univariate analysis show that secondary or higher level of education (55% vs 45%), high socioeconomic status (90.3% vs 53.7%), attending private hospitals (65.5% vs 34%), having diabetes for > 5 years (62.2% vs 37.8%), positive family history of DM  (69%), awareness regarding disease (80.4%) and use of pharmacological treatment i.e. (oral hypoglycemics, insulin or both) were significantly associated with SMBG adherence. Whereas gender, doctor consultation, life style modification (diet and exercise), diabetic complications, co-morbidities and HbA1c values showed no significance. Out of the patients who performed SMBG, 63.4% (n=210) made alterations to their treatment regimen (adjustment of insulin or skipping oral dose to avoid hypoglycaemia) and lifestyle (carbohydrate intake, physical exercise and consultation of physician for advice). Nearly 55.3% (n=183) of the patients using SMBG reported it helpful and believed that SMBG usage had a positive impact on their lives (*p*-value= 0.0001).

**Table 2:** Predictors of self-monitoring of blood glucose (SMBG) usage

CHARACTERISTICS	PARTICIPANTS (n (%))		* p*-value
	SMBG		
	Adherence	Non-adherence	
Gender			
Male	143/331 (43.2)	86/236 (36.4)	0.205
Female	188/331 (56.8)	150/236 (63.5)	
Education level			
Uneducated/ Primary education (grade 1-5)	149/331 (45)	176/236 (74.5)	<0.001
Secondary/Higher Education (grade 6-12/ graduate)	182/331 (55)	60/236 (25.4)	
Socio-economic status			
High (≥ 60,000 PKR)	65/72 (90.3)	7/72 (9.72)	<0.001
Low (< 60,000 PKR)	266/495(53.7)	229/495 (46.3)	
Hospital Setting			
Private	217/331 (65.5)	73/236 (31)	<0.001
Public	113/331 (34)	163/236 (69)	
Duration of diabetes			
< 5 years	125/331 (37.8)	130/236 (55.1)	<0.001
> 5 years	206/331 (62.2)	106/236 (45)	
Family history of diabetes	228/331 (69)	129/236 (54.7)	0.001
Awareness about diabetes	266/331 (80.4)	93/236 (39.4)	<0.001
Doctor consultation in			
< 3 months	203/331 (61.3)	162/236 (68.6)	0.073
> 3months	128/331 (38.7)	74/236 (31.3)	
Medications			
Diet and Exercise	11/331 (3.3)	13/236 (5.5)	0.203
Hypoglycemic tablets	169/331 (51.5)	194/236 (82.2)	<0.001
Insulin injections	53/331 (16.0)	12/236 (5.1)	<0.001
Both tablets and insulin injections	98/331 (29.6)	17/236 (7.2)	<0.001
Uncontrolled Diabetes (HbA1c > 6.5%)	66/82 (80.5)	32/40 (80)	0.091

**Figure 1:**
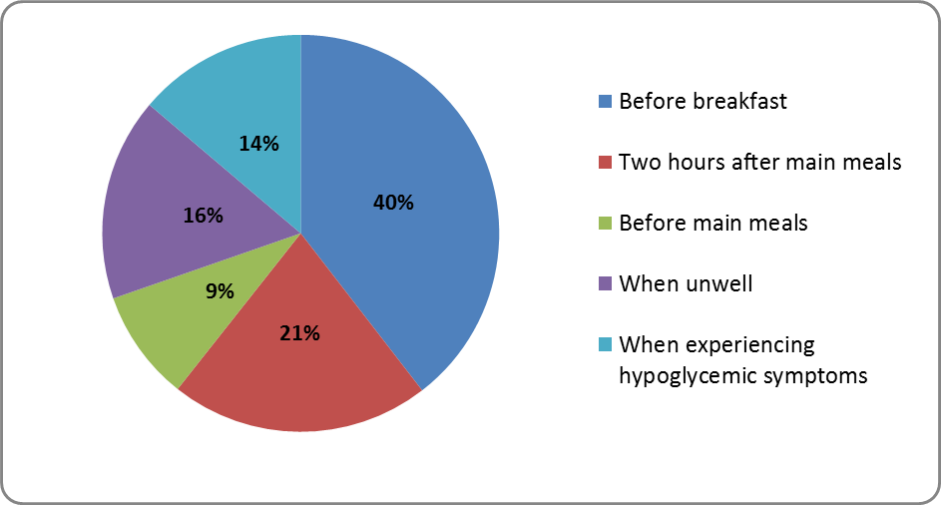
Frequency of blood glucose monitoring

Figure 1  shows 40% (n=132) of people performed self-monitoring before breakfast. Individuals who performed SMBG two hours after main meals were 21% (n=70). Only 9% (n=53) were reported to have checked their blood glucose before main meals. Meanwhile, 30% (n=175) of the population performed SMBG while feeling unwell (16%, n=53) and experiencing hypoglycemic symptoms (14%, n= 96). However, the relation of glycemic control of diabetes and the frequency of performing SMBG was insignificant (*p*-value= 0.212).

## Discussion

We found a relatively high prevalence (59%) of SMBG in our diabetic population. This corresponds to a study by Rafique, et al. [17], in which a majority of the population sampled from Karachi checked their blood glucose levels regularly. In contrast to our finding, a relatively low prevalence was observed in other developing countries such as Malaysia with a prevalence rate of 15.3% [7], 8.6% in Bangladesh [18] and 34% in Western Kenya [19].

We also observed that high adherence to SMBG was closely associated with financial stability and high education of the participants. These findings are consistent with those reported by a similar study conducted in Bangladesh [18]. In addition to these, patients having had diabetes for a longer duration and consulting the private healthcare sector were more likely to practice SMBG. No significant difference between the sexes was obtained regarding SMBG. This finding is in accordance with a similar research done previously in Karachi [20] but it is contraindicated by some other studies [18].

Our study reported that diabetes-related education sessions have a considerable impact on SMBG as 83% of the subjects who attended such sessions were monitoring blood glucose, out of which, 91.5 % were aware of complications of DM. This finding is in accordance with previous researchers enumerating significant patient-related factors contributing to SMBG underutilization [21]. Congruent with a previous study [22], we found that awareness about diabetes and its complications encourages better SMBG practices. As cited in the Journal of the American Diabetic Association (ADA), “patient’s SMBG related knowledge and skills” is a key criterion for effective SMBG [23]. Failure of SMBG in providing better glycemic control is an important predictor of neglecting SMBG practices as mentioned in another study conducted on Type 2 diabetics, which describes the most important reason for not using a glucometer i.e. “testing doesn’t help me control my diabetes” [24].

According to the ADA, SMBG is widely recognized as an integral part of treatment plans for patients on intensive insulin-based therapy [25]. We found that different treatment regimens directly influence the usage of glucometers. Also, consistent with a previous study [26], participants on oral hypoglycemic agents and/or insulin therapy were more adherent to SMBG as compared to those on non-pharmacological interventions (diet and exercise). This finding can be rationalized by the fact that patients on a proper treatment regimen were adamant on monitoring their progress while keeping a close check on their blood glucose level. Whereas patients using just diet and exercise do not religiously follow their plans and are often non-adherent.

In accordance with Parkin C, et al. [27], we found that a majority of the participants who used glucometers monitored their fasting blood glucose alone while failing to monitor postprandial glucose. This finding is significant because a previous study suggests that postprandial hyperglycemia confers an increased risk of cardiovascular disease and death [28] and a low incidence of this practice is worrisome. As already mentioned in the American Diabetic Association guidelines [14], we also observed that people with a low frequency of SMBG failed to adjust their treatment regimens.

Another secondary finding was that positive family history of diabetes proved to have a favorable impact on SMBG. Thus, it seems that prior exposure of the participants to the self-management/monitoring routine of their diabetic family members gave them an edge over those who were exposure-naive. High home-based SMBG has been observed in patients who could easily interpret the results of their glucometers and successfully apply the information obtained towards adjustment of the treatment regimen as compared to those who could not. This may be due to inadequate counseling provided to patients with regards to the optimal frequency and timing of SMBG, poor recording of resultant data and hence inability to interpret the results and failure to adjust their nutrition/diet and daily activities/exercise accordingly [24].

A little less than half of the sample population reported not using glucometers at home. This could be because a majority of uneducated diabetic patients lack proper knowledge about the significance of their use and interpretation of glucometer readings. Based on the aforementioned findings, our study failed to identify a significant impact of SMBG in the lives of diabetics possibly due to lack of access to guidelines, specifically suitable for the Asian health sector. Consequently, people from third-world countries like ours find it hard to adhere to Western SMBG guidelines due to multidimensional differences in health care facilities [23]. In accordance with previous studies [18], our results also reported certain other factors contributing to low adherence of SMBG; for example, the high cost of monitoring supplies (particularly strips) made glucometers too expensive and burdensome for regular use, particularly in households with low family income. During our data collection, we came across a few private healthcare setups which provided their regular registered patients with free SMBG toolkits that clearly resulted in a greater number of patients performing SMBG. Therefore, we suggest that the government health care sector provide patients visiting public hospitals with SMBG toolkits and that the out-of-pocket cost of a glucometer along with its strips should be reduced. This practice has also been observed in other countries as well [8].

Consequently, substantial efforts aimed at providing serial diabetes education dissemination to patients belonging to all walks of life should be developed. That includes systematically clarifying ambiguities related to the practice of SMBG and counseling the patients with special instructions on how, when and why to test. Keeping in mind the heterogeneity of languages spoken across Pakistan, multilingual teaching resources should be administered, be it education sessions, brochures, electronic or print media. In addition to that, patients should be trained to interpret readings for blood glucose. Low adherence is likely to occur when behaviors are not directly supervised [29], hence SMBG techniques, results and ability to use SMBG data to adjust therapy should be evaluated at regular intervals in order to accustom the patients to recommended usage protocol. We recommend special guidelines to be devised targeting the population of Pakistan keeping the American Diabetic Association's guidelines as a reference [19]. Our study shows that Pakistan is inching towards achieving an optimal SMBG usage among its masses and a continuous serial approach encompassing these suggestions should be taken into consideration to help speed it up.

There are certain limitations in this study that need to be considered. Firstly, our study sample was confined to the diabetic population of Karachi only, so we cannot generalize our findings to and implement the recommendations on rural areas because of their low literacy rate and overall low income. Secondly, the frequency of glucometer usage was self-reported by the patients which may not be reliable and might have some recall bias. Since most of our data were collected from outpatient settings where the patient influx is very high, the average patient-interviewer time was limited. As a result of which, we were unable to measure their heights and weights to subsequently incorporate the body mass index (BMI/ kg/m^2^) values in our study and thus failed to show the association of obesity with SMBG usage. Lastly, while our study was able to identify the penetration of SMBG techniques into the local population and factors that influenced it, we were unable to gather data which would reflect the competence of individual patients to effectively interpret glucometer readings in order to work towards achieving long-term treatment goals. We also did not measure how much of technical training patients received at baseline in order to familiarize them with the equipment. Future endeavors in this area would do well to incorporate these measures into their studies.

## Conclusions

Our study demonstrates the increasing trend in use of SMBG. Diabetic patients with a higher socioeconomic status and education level were more likely to practice SMBG. Lack of awareness and cost of glucometers were reported to be the main reasons for not practicing SMBG. Given these factors are easily modifiable, government subsidized initiatives and awareness programs can result in a successful public health strategy to control diabetes.

## Appendices

QUESTIONAIRE 

Age:   _____________                                

Gender:   ____________

Occupation:  ___________________                  

Ethnicity: 

a) Sindhi      b) Punjabi      c) Balochi      d) Pathan      e) Urdu Speaking      f) Other

Marital status: 

a) Married         b) Unmarried       c) Divorced

Q1. Education Level:

        a) No formal education       b) Primary         c) Matric         d) Intermediate       e) Higher Education

Q2. Monthly income:     

        a) Below 5000Rs       b) 5000Rs-30000Rs      c) 30000rs-60000Rs      d) 60000Rs-1 Lac Rs                                                  

        e) More than 1 Lac Rs

Q3. Smoking History:       

        a) Never Smoked           b) Used to smoke          c) Sometimes           d) Everyday

Q4. Physical Activity:     

        a) Yes          b) No         c) Occasionally

Q5. Which hospital did you visit for consultation?

        a) Government hospital     b) Private hospital

Information about Diabetes:

Q6. Duration of diabetes:     

        a) <1year   b) 1-5 years   c) 5-10 years   d) More than 10 years

Q7. Do you have a family history of diabetes?   

       a) Yes      b) No

Q8. How often do you consult your Doctor/G.P for your diabetes?  

        a) Every 3 months   b) Every 6 months   c) >12 months    d) Never

Q9. Current diabetes treatment regime:   a) Non-pharmacological   b) Tablet         c) Tablet and (diet and exercise)           (oral anti-         insulin injection diabetic agent) d) Insulin injection only

Q10. Any diabetes complications e.g. eye problem, feet problem, kidney problems etc? a) Yes               b) No               c) Not sure      Please state: ____________________

Q11. History of hospitalization secondary to diabetes and its related problems: a) Yes               b) No              

Q12. Have you attended any diabetes education session?

         a) Yes               b) No                                                                                                                                                                 

Q13. Do you have any awareness about the factors leading to diabetes and its complications?

          a) Yes               b) No              

Q14. Is your diabetes well controlled?

          a) Always in control    b) Often in control    c) Sometimes

Q15. Do you use a gluocometer?

         a) Yes               b) No            

Q16. If no, select your reason: a) Discomfort   b) Not properly educated about usage   c) No awareness about glucometer                    

          d) Expensive

If yes, then answer the following questions:

Q17. Why did you start using a glucometer?

          a) Hospital/Primary Health Care Centre    b) Mass Media    c) Friends/Relatives  

Q18. Duration of use: _________________

Q19. Frequency of self monitoring of blood glucose in a week?

         _______________ Times per week

Q20. Time of self blood glucose monitoring: (may select more than one answer)

           a) Before breakfast   b) 2 hours after   c) Before main meals   d) When unwell   d) When main meals                                                                          experiencing hypoglycemic symptoms

Q21. Do you record your glucometer readings?

         a) Yes               b) No

Q22. Adjustment of diabetes treatment based on the blood glucose results?

         a) Yes               b) No              

Q23. Do you share your glucometer/strips?

          a) Yes               b) No

Q24. Does the use of glucometer have any impact on your daily life?

         a) Yes               b) No

Q25. Do you have any other co-morbidity?

          a) Hypertension    b) High Cholesterol    c) Obesity    d) Other, please state ____________
